# Tri-State Medical Society or Alabama, Georgia and Tennessee

**Published:** 1891-12

**Authors:** 


### TRI-STATE MEDICAL SOCIETY OF ALABAMA, GEORGIA AND TENNESSEE.

### THIRD ANNUAL MEETING, HELD IN CHATTANOOGA, OCTOBER 27, 28, AND 28.

#### FIRST DAY.

Society called to order by Vice President B. T. CAMP.

Opened with prayer by Rev. D. Vance Price.

The Committee on Necrology offered a set of resolutions on the deaths of Drs. T. F. Cary of Florida, W. B. Wells and W. P. Craig, both of Chattanooga, which were adopted.

After the reports of committees and the transaction of some miscellaneous business, Dr. S. S. Kerr read a paper reporting a case of "Neuro-Mimetic Hip" trouble, and presented the patient.

This was a case in which the diagnosis of gonorrhœal rheumatism had been made, but he was unable to see the case in that light. The nervous system and the family history indicated a nervous element, and there was an hysterical element in the case. A partial cure was effected by suggestion, but the patient still walked on his toe, for which he could see no reason, as there was no shortening nor tenderness about the hip, or other signs indicating organic disease.

DR. TRIPPE said that he had treated the case before Dr. Kerr. The patient had gonorrhœa four weeks before he saw the case. There was increased temperature, 102° to 104°. There had been two marked chills and a typical picture of gonorrhœal rheumatism, although an hysterical element was recognized in the case.

DR. REEVES thought the case one of involvement of the cord, in which there was an attack of gonorrhœa, and that this set up a new train of reflexes. He called attention to the fact that every discharge from the meatus was not a gonorrhœa, and as a test he stated that the discharge from a specific case was acid, from a non-specific alkaline.

DR. J. B. COWAN thought that the history as given by Dr. Trippe indicates some specific trouble, and if most of the members had seen the case it would have been diagnosed as gonorrhœal rheumatism. Where we have such a specific trouble and a history of masturbation as here, we would expect some hysterical symptoms. A man may have an hysterical joint just as a woman may have the globus hystericus.

DR. DRAKE, from the history and an examination, thought the case one of gonorrhœal rheumatism.

Whenever there is a pain there must have been a cause, past or present.

DR. KERR had nothing to say as to the condition before he saw the case. He agreed that there were neurotic symptoms. It was a difficult task to make a diagnosis. He had brought the patient so as to find out how to make him stop walking on his toe, for which he could see no reason.

### AFTERNOON SESSION.

President ROBERT BATTEY presided.

Dr. E. T. CAMP of Gadsden, Ala., read a paper on

### THE SUMMER DIARRHŒA OF CHILDREN, in which he gave as the causes, 1st, improper food; 2d, high temperature; 3d, micro-organisms. In some cases there was a neurotic element. He reported one case where the diarrhœa was cured by circumcision, there being no change in the other treatment.

DR. BATTY asked if any of the members had any experience that would confirm the views of the writer that the prepuce might keep up the diarrhœa.

DR. GAHAGAN had a case of persistent diarrhœa in which there was an elongated prepuce. He would circumcise the case and report next year.

DR. COWAN had not noticed that male children were more subject to diarrhœa than females. There was often fault in the diet, both as to quality, and especially quantity.

DR. BERLIN called attention to the fact that the Jewish children have diarrhœa as frequently as the Gentiles, and could see no connection between a stomach loaded with bacteria and an abnormal prepuce.

DR. J. L. ATLEE confirmed the experience of Dr. Camp. He had seen cases in which, after circumcision, the diarrhœa began to improve with no change in the other treatment.

In closing Dr. Camp said that he had reported but one case in his paper, but that he had seen a number of others in which there was a like result.

DR. GEORGE WILEY BROOME, of St. Louis, read a paper on

### REPORT OF A SUCCESSFUL CASE OF KOLPO-HYSTERECTOMY, INCLUDING A BRIEF REVIEW OF THE PRESENT STATUS OF THE OPERATION.

in which he advocated the operation in all cases of epithelioma or carcinoma of the cervix, or of the body of the uterus, regardless of the extent of the disease. Amputation of the uterus should never be performed.

DR. DAVIS' experience had been that these cases when sent to him were too far advanced to justify an operation. He had not been convinced, where but a limited part of the cervix was involved, that an amputation was not as good as the radical operation. Many cases were morphine eaters, and the condition of the intestinal tract was one of importance.

DR. BERLIN thought the total extirpation was better than high amputation. When the disease had passed beyond the uterus it was to late to amputate in any way.

DR BATTEY had grave doubts as to the advisability of the operation. In the early stage the diagnosis was difficult. In some of the cases sent him as cancerous, cures were effected by the application of iodine, etc. Of the cases cured he had grave doubts as to the diagnosis.. On the other hand there were many deaths after the operation, if not immediately, within a short time. As in the case of Sen. Hill, many will not consent to an operation until a malignant growth has advanced beyond the stage when it can be removed.

DR. KEY preferred the clamp to the ligature. Early diagnosis is of importance, and this can only be made by an expert pathologist, and as soon as made the uterus should be removed.

NIGHT SESSION.

Addresses of welcome were made by Dr. J. R. Rathmell, President of the Chattanooga Medical Society and Col. Garnett, Mayor of the city.

DR. ROBERT BATTEY responded on behalf of the society.

DR. GEORGE E. WEST reported

TEN CASES OF LAPAROTOMY, with one death. Three were for the removal of diseased ovaries and tubes, one for the cure of oöphoro-epilepsy, one for the removal of ovarian cyst, three for the relief of symptoms caused by uterine fibromata, two exploratory incisions. Of the nine recoveries six were perfect cures, three partial cures from incomplete operations.

DR. DAVIS said that it was the improved technique that gave success in these operations, which required not only book knowledge, but also special training.

DR. BROOME advocated early operation. He insisted on sterilizing the instruments, and indorsed Arnold's sterilizer. Morphine should never be given after a laparotomy.

DR. REEVES felt grateful to the author for his remarks on conservatism. He quoted Dr. Mitchell, who said that in his experience he had sent thirteen cases to the surgeon. Five of these were not improved. Dr. Gardner had said that the majority of cases operated on were not any better five years after the operation.

SECOND DAY.

Opened with prayer by the Rev. J. W. Bachman.

After some miscellaneous business Dr. Robert Battey addressed the Association on

OVARIOTOMY; ITS USE AND ABUSE.

He said that the fundamental idea in the operation he had devised was to produce rest. The difficulty of curing many chronic diseases lies in the fact that rest is an impossibility, as with the heart rest means death. Rest is an impossibility to an ovary.

The objects of the operation are—

1st. The prolongation of life. Years ago Sir Spencer Wells said that he had added 3000 years to the sum of human life; now it is probably double that.

2d. The restoration of a disordered mind. There is a prejudice against the operation, owing to the fact that cases have not been properly selected, and alienists want the ovariotomist to cure their cases after they have exhausted every other means of cure, when it is often too late.

DR. GOODELL asserted that an insane woman had no business with children.

DR. BATTEY would hardly go so far.

3d. The cure of epilepsy. As in the case of insanity, there should be some connection between the epilepsy and the ovaries. It does not follow that because a woman has epilepsy that her ovaries should be removed. Here Dr. Goodell had good results.

4th. The relief of intolerable pain. Especially when the pain has a tendency to produce that detestable habit, opium eating, a habit little short of insanity. Where the habit has been formed the operation will cure the case if the woman can break the habit.

One of the abuses of the operation is to perform a single operation for the sake of the notoriety it would bring. This ought to be a specialty as much as the eye. Success depends on the skill of the operator, which can only come from experience. It depends also on native ability, and every man should study his natural talents in the light of statistics, and choose the field where he is most successful.

The operation of ovariotomy to stop child-bearing is a detestable practice. The operation should never be done without ample consultation, first, to protect the physician; second, in the interest of the profession at large; third, in the interest of the patient.

DR. DAVIS thought that as much could be done by simply incising the muscle as by a normal ovariotomy. The operation has no place in the treatment of nervous diseases.

DR. BROOME suggested that as it was well known that ovariotomy produced atrophy of fibroid tumors by cutting off the blood supply, therefore ligation of the uterine artery might produce as good results.

DR. WILSON advocated the operation in cases of mania; did not believe that insane women should have children.

To confirm Dr. Battey's views, Dr. Cowan reported a case of epilepsy cured by the operation.

DR. BATTEY, in closing, gave the indications for the operation, as follows:

1st. The case must be desperate. 2d. It must be incurable by ordinary means. 3d. There must be a reasonable hope of cure.

In the last two years he had advocated the removal of senile, diseased ovaries for the cure of insanity, citing cases.

DR. W. E. B. DAVIS, of Birmingham, Ala., read a paper entitled

TREATMENT OF INFLAMMATION ABOUT THE HEAD OF THE COLON, in which he said that cases must be selected for the operation; important symptoms must not be masked by the administration of opium. More reliance should be placed on regional tenderness than on the temperature. An inflammation about the head of the colon is always an appendicitis, the involvement of surrounding tissues being secondary. Early operation is necessary.

DR. CUNNINGHAM was of the opinion that the whole question should be rewritten. The peritoneum is always involved to a limited extent.

DR. SHIMWELL said that the temperature may not be increased, and related a case confirming the statement. There is no rule when to operate; each case must be judged on its own merits.

DR. KARL VON RUCK, of Asheville, N. C., read a paper on

THE CURE OF TUBERCULOSIS ON THE PRINCIPLE OF NUTRITION, in which he said that the diagnosis with the microscope could not be made in the early stage. No one measure should be relied upon in the treatment. He was surprised that greater harm had not been done by the large doses of tuberculin that had been used. In the early stage the treatment was often inefficient, when the cases could be cured. Climate was of importance, and all measures that could benefit the patient should be employed.

DR. REEVES advised the use of the microscope in all cases to confirm the diagnosis; if it be not tuberculosis it is syphilis. Primarily the disease is due to lymph stasis.

DR. VON RUCK called attention to the fact that in the early stage there is no sputum and no bacteria, so that the diagnosis cannot be made with the miscroscope.

DR. J. C. SHAPARD, of Winchester, Tenn., read a paper on

MILK SICKNESS, stating that the disease existed only in a limited area; that it was contracted from the cow. The poison seemed to be neither animal nor vegetable, but mineral. The disease called trembles in the cow resembled lead poisoning in man.

DR. COWAN said that the subject was of so much importance that the Government had offered a reward for the discovery of the cause. He had seen one case, and thought at first that it was one of lead or cobalt poisoning.

DR. REEVES said that the bacteria had been found; that they were spirilli, for which quinine was the best remedy.

DR. J. B. MURFREE, of Murfreesboro, Tenn., read a paper on

THE NECESSITY FOR ASEPSIS IN PRIVATE OBSTETRICAL PRACTICE.

He advanced the idea that it was more necessary to protect the wounded surface here than in an open wound. The decreased mortality in hospital practice he thought due to the use of antiseptics. In private practice cleanliness was necessary, and sometimes antiseptics ; especially should the hands be clean, and the examinations be as few as possible.

DR. BAXTER indorsed the paper in the main, but thought that in private practice the danger of infection was ten times as great as in hospital practice. The nurses should be watched, as they know nothing of surgical cleanliness.

DR. SHIMWELL thought the injury to the mother was a factor in these cases that was overlooked. Wherever there had been a *post mortem* great injury to the tissues had been found.

DR. GOWAN thought the great secret was cleanliness, but that antiseptics have their place.

In a large number of cases observed Dr. Wilson had not found the results any better with antiseptics than with simple cleanliness with sterilized water. The results were as good where the patients were aggregated as where they were segregated. Vaginal irrigation was not necessary, for the cases did as well by simply washing the vulva.

DR. CUNNINGHAM thought with Dr. Shimwell that the result was often due to traumatism. He always uses the Credé method of expelling the placenta.

NIGHT SESSION.

At 8 P. M. an elegant reception was tendered the members at the residence of Mr. and Mrs. M. R. Wilson, 229 East Terrace.

THIRD DAY.

MORNING SESSION.

Opened with prayer by Rev. Robt. J. Willingham.

DR. N. G. BOGATR, of Chattanooga, read a paper on

LACERATED CERVIX.

He advocated the operation only when there were troublesome symptoms produced by the laceration, and other measures fail.

He described the operation mainly as laid down by Skeene. The cause of failure was imperfect preparation of patient, imperfect operation or imperfect after treatment.

DR. CAMP said that it was necessary to remove all the cicatricial tissue; silver sutures the best; douches not necessary. He does not indorse the use of ergot after delivery.

DR. DAVIS said the paper presented the present status of the operation. The condition requiring it could be prevented by proper attention after confinement. Ergot is of use after confinement, not only to cause contraction of the uterus, but it also closes the mouths of the small vessels and lessens the danger of septic poisoning. He examines all of his patients six weeks after confinement if possible. In subinvolution the faradic current is of value, despite the assertions of many that electricity is of no use in gynæcology.

DR. REEVES had gotten good results in these cases by supporting the womb with a Fowler pessary, and had cured some by this means. He gave minute doses of ergot after confinement.

DR. BOGART, in closing the discussion, said that he gave support to the uterus in these cases, but that he preferred to do this with medicated lamb's wool tampons, instead of using a hard rubber pessary.

DR. G. W. DRAKE, of Chattanooga, presented a paper on

THE PHYSIOLOGY AND CHEMISTRY OF THERAPEUTICS.

In this he maintained that the infectious diseases are caused by ptomaines or toxines evolved by bacteria in the body. He proclaimed that "chemical antagonism" was the safest, the most scientific and most rational means of cure, rather than that of "physiological antagonism." He argued that all bacterial toxines had an antidote for which we should look. The tendency was to return to specific medication along more scientific lines. The age demands rational medicine.

DR. PURDON called attention to the fact that the antiseptics were used thirty years ago empirically, for he had used the permanganate of potash in cholera; he had also used the peroxide of hydrogen.

DR. B. T. SHIMWELL, of Philadelphia, read a paper on

ARTIFICIAL ANUS VS. ANASTOMOSIS.

DR. JOHN E. PURDON, of Cullman, Ala., read a paper on

THE CONSERVATISM OF ENERGY IN MODERN PHYSICS, in which he claimed that in the face of established facts of mental and physical action at a distance, nothing was left to the physiologist but to acknowledge the existence of an extra muscular mode of the externalization of energy in relation with conscious or sub-conscious will and design. He held the opinion that the ether of space had its physiological as well as its physical side, and that, as the reservoir of the work-doing power of the universe, it bore a relation to the Universal Life analogous to that which the blood and nervous system held to the individualized spirit. He based his theory of an ethereal nervous medium upon the results of his own sphygmographic researches which showed the similarity of the pulse traces of individuals *en rapport* during extraordinary manifestations of energy, such as "knockings" and "telepathic influence." Dr. Purdon deposited publicly with the secretary the photographs of a selected set of pulse tracings, taken by himself, in illustration of the above view, and claiming the absolute originality of the method for himself.

DR. COWAN said that the grandest result of energy was thought, by the arrangement of matter, by the correlation of force we have this power. This we derive from solar force.

DR. CUNNINGHAM thought that we know nothing about the matter.

DR. DRAKE took issue with Dr. Cowan, that the original force was solar, for energy existed before the sun was made, and came from the Deity. To this Dr. Cowan assented.

DR. J. P. STEWART, of Attalla, Ala., read a paper on

EVOLUTION FROM A SCIENTIFIC STANDPOINT, in which he advocated the doctrine from scientific considerations.

DR. DRAKE said that the reproductive energy in the human ovum was the unseen hand of God moulding its protoplasm into perfect form.

DR. PURDON said that man belongs to a different class from the lower animals. Evolution is true as a formula, as a partial formula.

AFTERNOON SESSION.

DR. HENRY WM. BLANC, of Sewanee, Tenn., gave his experience in

A REVIEW OF FIVE YEARS OF DERMATOLOGICAL PRACTICE IN NEW ORLEANS.

He reported 2,013 cases seen in public and private practice. Twenty per cent. were eczema, elsewhere the per cent. is thirty or thirty-five. Epithelioma in the form of rodent ulcer figured conspicuously in the report. A large number of leprosy cases were reported. Many of these cases were of foreign birth, or children of foreigners. The author believes in the contagiousness of leprosy, but thinks that in many of his cases the disease was contracted from some animal source, as in eating raw meat, or in preparing meat for the table.

DR. R. M. CUNNINGHAM handled the subject of

CROUPOUS PNEUMONIA.

A paper was read by Dr. Y. L. Abernathy, of Hill City, Tenn., on

DOCTORS.

DR. W. P. McDONALD, of Hill City, read a paper entitled

LEGISLATION, which was not discussed, as it dealt with matters of a political nature.

NIGHT SESSION.

DR. W. C. TOWNS read a paper on

ANGINA PECTORIS, in which he gave as the conditions in the disease 1st, pseudo-angina pectoris; 2d, that form in which there is sclerosis of the coronary arteries; 3d, where there is valvular disease. The treatment depends on the cause. In the first form we have a neurosis, and we correct anything we find at fault with any of the organs; secondly, we give tonics, potassa iodide, arsenic, nitrites, thirdly, we prescribe during the attacks such drugs as amyl nitrite, chloroform and opium.

DR. DRAKE thought angina pectoris a symptom, rather than a disease, sometimes the result of organic lesions, but often merely a cardiac neuralgia. He uses nitro-glycerine with atropia for the pain.

DR. PURDON gives as routine treatment the salicylate of soda where it is caused by cold (lowering of temperature). This is combined with strophanthus to prevent relapse.

DR. CAMP believes it to be due to a rheumatic diathesis, and uses chloroform by inhalation.

DR. WERT would be afraid to give chloroform owing to the pathology.

DR. PURDON said that by no means must electricity be used.

DR. BAXTER did not think chloroform specially dangerous, and cited cases.

DR. CUNNINGHAM believes in giving atropia and nitrite of amyl. He did not consider an intermittent pulse to contraindicate chloroform.

DR. TOWNS closed by saying that he did not lay much stress on the above treatment.

### NIGHT SESSION.

DR. E. H. KUYKENDALL, of Chattanooga, read a paper on

### BROMIDE OF ETHYL AS AN ANÆSTHETIC, advocating its value and safety when given for short operations (one minute) and in dose of not over a dram. It is given free from air, anæsthesia is complete from one half to one minute. The effects last about two minutes, when the patient wakes as from a natural sleep. Nausea is seldom produced.

DR. DAVIS said that one accustomed to give ether was not safe to give chloroform, and it might be so with this ; the deaths may have been due to faulty administration. Nitrous oxide was a rapid anæsthetic, and was considered the safest.

DR. SMITH suggested that if the doctor would give the number of cases observed by him that it would be of interest.

DR. BERLIN said that an objection was the odor. He related two cases of death from the drug.

DR. GAHAGAN asked Dr. Kuykendall for the mortality, how anæsthesia was produced and the antidote.

DR. KUYKENDALL replied that there had never been a fatal case unless the administration was prolonged. Dr. Chisholm had used it in three hundred cases without a bad result. So far as he knew there had been but two deaths. He did not know how it kills, or how it produces anæsthesia. The antidote is the same as in threatened death from chloroform.

DR. WILLIS WESTMORELAND, of Atlanta, discussed

BRAIN SURGERY, saying that the surgeon had gone into the brain where the physiologist had said that he could not go. An exploratory incision into the brain substance was just as justifiable as in laparotomy. In abscess and tumors there has never been a cure without operation; where the incision has been thorough the results have been good. The safeguard is antisepsis, without which there is uncertainty. In operating, the ventricles must be avoided.

DR. DRAKE argued that surgeons had never gone farther than the physiologist had mapped out for them. They dare not invade the fourth ventricle in the vicinity of the respiratory center.

DR. WESTMORELAND reminded Dr. Drake that it was not due to the physiologists, but to the fact that some years ago a man had recovered after a crow-bar had gone through his brain.

DR. BERLIN related a case of insanity coming on after an injury to the skull cured by the same means.

DR. CRUMLEY believed that all functions were localized. Some areas can be invaded, others cannot.

DR. CUNNINGHAM said that most of these cases would die without operating, and that the surgeon was justified in doing anything that offered the least hope.

DR. STEWART reported a case of brain surgery where the whole frontal bone was taken away.

DR. WESTMORELAND said that to Dr. Briggs was due the credit of being one of the first to do this work. The success depends largely on drainage, and it may be necessary to make a counter-opening.

A paper by W. L. Bullard, of Columbus, Ga., was read asking

SHOULD NOT OCULISTS BE MORE CAREFUL IN PRESCRIBING COLORED GLASSES?

in which he showed that the smoked glasses were generally better than the colored glasses, and that there was a more serious objection to the curved glasses, for the reason that they possessed some refractive power when we wanted a plain glass.

The following are the officers for the ensuing year:

President, W. E. B. Davis, Birmingham, Ala. Vice-Presidents, D. H. Howell, Atlanta, Ga.; J. C. Shapard, Winchester, Tenn.; J. P. Stewart, Attalla, Ala. Secretary, Frank Trester Smith, Chattanooga, Tenn. Treasurer, B. S. Wert, Chattanooga, Tenn. Recorder, W. L. Gahagan, Chattanooga, Tenn. Councillors, J. B. Murfree, A. B. Frix, John E. Purdon, G. W. Drake, J. W. Clements, E. T. Camp.

---

We regret to note that our esteemed picturesque contemporary, the *Dixie Doctor*, which has boasted of being so lively, and robust, and healthy, and vigorous, has heen suffering with some catamenial irregularities of late. She has missed some of her monthly periods, and her menstrual functions seem to be getting deranged. Her last appearance, the October *issue*, came "past the time," and was much diminished in volume. Can it be that one so young, at one time so promising, at all times so hopeful, should be slowly undergoing her "change of life" so soon? We hope that the gentleman who directs the functions of the *Dixie Doctor* will apply the suitable remedies to establish a normal equilibrium.

---

Another epidemic of the influenza has broken out among the Russia peasants. It is now prevailing in Russia and Germany.

